# Applying the Atomic Force Microscopy Technique in Medical Sciences—A Narrative Review

**DOI:** 10.3390/biomedicines12092012

**Published:** 2024-09-03

**Authors:** Karolina Krawczyk-Wołoszyn, Damian Roczkowski, Adam Reich, Magdalena Żychowska

**Affiliations:** 1 Doctoral School, University of Rzeszow, 35-959 Rzeszów, Poland; karolinakrawczyk10@wp.pl; 2 Department of Dermatology, Institute of Medical Sciences, Medical College of Rzeszow University, 35-959 Rzeszów, Poland; droczkowski99@gmail.com

**Keywords:** atomic force microscopy (AFM), biomedical research, melanoma, oncology, skin cells

## Abstract

Penetrating deep into the cells of the human body in real time has become increasingly possible with the implementation of modern technologies in medicine. Atomic force microscopy (AFM) enables the effective live imaging of cellular and molecular structures of biological samples (such as cells surfaces, components of biological membranes, cell nuclei, actin networks, proteins, and DNA) and provides three-dimensional surface visualization (in X-, Y-, and Z-planes). Furthermore, the AFM technique enables the study of the mechanical, electrical, and magnetic properties of cells and cell organelles and the measurements of interaction forces between biomolecules. The technique has found wide application in cancer research. With the use of AFM, it is not only possible to differentiate between healthy and cancerous cells, but also to distinguish between the stages of cancerous conditions. For many years, AFM has been an important tool for the study of neurodegenerative diseases associated with the deposition of peptide amyloid plaques. In recent years, a significant amount of research has been conducted on the application of AFM in the evaluation of connective tissue cell mechanics. This review aims to provide the spectrum of the most important applications of the AFM technique in medicine to date.

## 1. Introduction

Modern medicine aims to study single cells, chemical molecules and signaling pathways to understand pathological conditions as deeply as possible. Penetrating deep into the cells of the human body in real time has become increasingly possible with the implementation of modern technologies into medicine—such as atomic force microscopy (AFM). AFM is a type of microscopy that utilizes a scanning probe (Scanning Probe Microscopy—SPM). It allows for the possibility of achieving a resolution of fractions of a nanometer, or even on an atomic scale [1,2]. A lateral spatial resolution for an extracted and immobilized cell membrane patch is approximately 1 nm [3,4], while the resolution for surfaces of living cells can approach ~10 nm [5,6]. The vertical resolution in the Z-plane is about 0.01–0.1 nm, which constitutes one of the main advantages of this technique [7,8]. AFM enables the effective live imaging of the cellular and molecular structures of biological samples (such as cellular surfaces, components of biological membranes, cell nuclei, actin networks, proteins, and DNA) and provides three-dimensional visualization of the surfaces, in the X-, Y- and Z-planes. Testing may take place in a variety of environments (vacuum, air or liquid) [9,10,11,12,13,14,15]. Typical images reach 10–100 µm in the X- and Y-planes and up to 10 µm in the Z-plane [9,10].

The AFM method has also been used to study the nanomechanical, electrical and magnetic properties of cellular structures [3,9,16,17,18,19,20]. By examining the mechanical properties of the sample, the stiffness/elasticity, as well as the viscous and adhesive properties of the sample’s surface, can be determined, and a tensile or compressive modulus (Young’s modulus) can be created [8,9,18,19,21,22,23,24,25,26,27,28]. The AFM is now evolving into a multi-parameter sensing device, but it originally functioned as a tool for imaging and direct force measurement [29,30].

The spring constant of the cell surface is influenced by extracellular and intracellular factors and can provide information about changes in the cell, e.g., it may be used to evaluate the effect of antibiotics on bacterial cells or the impact of mutations on eukaryotic cells [2,9,14,22,31,32,33,34,35,36,37,38]. In addition, it is possible to measure interaction forces between various biomolecules. The ability to measure the interaction forces between the AFM’s scanning tip and the sample provided an opportunity for the use of AFM in force spectroscopy [29,30]. Binding forces between complementary oligonucleotides and interactions in receptor–ligand complexes or between adhesion proteins were all studied using AFM [39,40,41,42]. Attaching a specific molecule probe to the AFM tip enables selected molecules of interest to be found; these specifically interact with the probe in the studied sample. Such evaluation is possible thanks to molecular recognition force microscopy (MRFM) or topography and recognition (TREC) microscopy [9,43,44,45,46].

The main advantages of AFM are that there is no need for the staining, fixation, dehydration or labeling of samples [8]. Scanning probes can image a variety of materials without destroying them—from delicate, soft biological samples to titanium oxide–metal surfaces [47]. AFM may also be combined with other techniques, such as single-cell force spectroscopy (SCFS), single-molecule force spectroscopy (SMFS), chemical force microscopy (CFM), and atomic force microscope-infrared spectroscopy (AFM-IR), which only extends the potential applications for this method [1,17,48].

## 2. Construction of the Microscope, the Physical Basis, and the Principle of Its Operation

Unlike classical optical microscopy, in AFM, representing the scanning probe microscopy (SPM) group, measurement is performed by the deflection of the cantilever with the probe attached due to contact with the surface or intermolecular forces [49]. The tip is moved along the surface of the tested material. The forces of interaction of the tip’s atoms with the surface are measured by recording the movement of a cantilever, which does not exceed 1 nN. Using a set of electronic sensors, information about the deflection of the cantilever from the equilibrium position is recorded, giving information about the force of interaction between the tip and the sample [50]. The principle of the AFM can be illustrated by van der Waals forces [51]. The interaction energy between a pair of neutral atoms, located at a given distance, also called the Lennard-Jones potential (*V*), is defined by the following equation:

$$V\left(r\right)=4\varepsilon \left[{\left(\frac{\sigma }{r}\right)}^{12}-{\left(\frac{\sigma }{r}\right)}^{6}\right]$$

where *r* is the distance between two atoms, and ε and *σ* are the bond energy and bond length, respectively [52].

**Figure 1:**
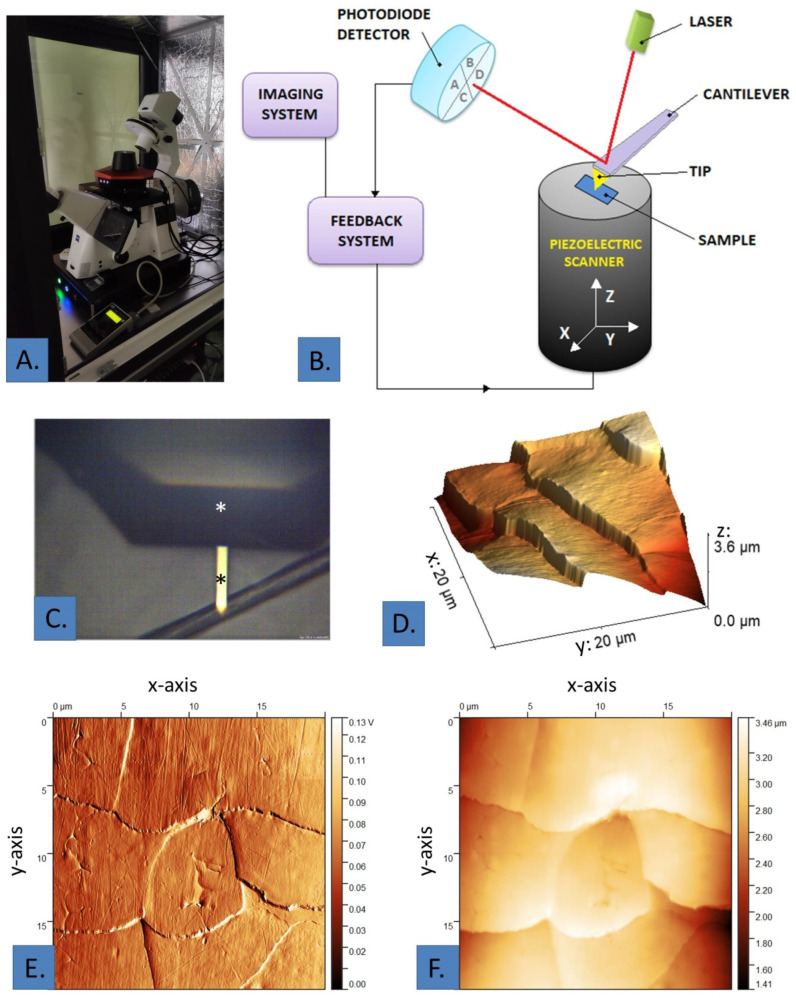
(**A**) Photograph of the atomic force microscope; (**B**) schematic of the AFM construction. TIP: tip of the cantilever. (**C**) cantilever (white asterisk) with AFM tip (black asterisk) scanning hair—optical microscope view; (**D**) AFM graphic of the hair surface—3D projection: x—the dimension transverse to the longitudinal axis of the hair fiber; y—the dimension parallel to the longitudinal axis of the hair fiber; z—height of hair surface; (**E**) AFM image of the hair surface—deflection image: x—the dimension transverse to the longitudinal axis of the hair fiber; y—the dimension parallel to the longitudinal axis of the hair fiber; (**F**) AFM image of the hair surface—*Z*-axis image: x—the dimension transverse to the longitudinal axis of the hair fiber; y—the dimension parallel to the longitudinal axis of the hair fiber.

The diagram below (Figure 1B) shows the operation of the AFM. The laser generates a beam of radiation, which is directed at the top surface of the cantilever and is then reflected from it. The reflected laser beam is collected by a split photodiode [53]. The cantilever is mounted on a piezoelectric scanner that allows movement in the horizontal (x, y) or vertical (z) dimension. The geometry of the scanner is controlled by the value of the applied voltage [54]. A flexible cantilever, consisting of a base, a micro spindle and a sharp cone- or pyramid-shaped tip, is used to interact with the sample. Probes are usually made of silicon, silicon dioxide or silicon nitride, created during nanofabrication processes [55]. By approaching the probe to the sample for a short distance, its deformation resulting from the interaction between the tip and the surface of the test substance is observed. Measurements using AFM are designed to obtain information about forces, so the deflection must be converted to a force (F) of interaction according to the principles of mechanics and Hook’s law, which states that the magnitude of the force acting on the tip of the sample is proportional to the deflection of the arm:

F=k×x

where *x* is the deflection of the cantilever and *k* is the elasticity constant of the cantilever. Deflection (*F*) is measured in volts [*V*] converted to units of force [*N*—newton] using the following formula:

$$F\left[nN\right]=I\left[V\right]\times invA\left[\frac{nm}{V}\right]\times k\left[\frac{N}{m}\right]$$

where *I* is the signal recorded in the microscope [*V*], *invA* is the sensitivity of the photodetector, *k* is the elasticity constant, *m*—meter, and *n*—nano [56].

The resolution of AFM depends on several factors. These include the radius of curvature of the scanning tip and the precision of the entire scanning system, as well as the nanopositioning system [57]. AFM uses a positioner to place samples under a cantilever with a sharp probe to manipulate objects at the nanoscale. When measuring a sample, the XY nanopositioner is used to move the sample across an area in the system while the Z nanopositioner allows for the positioning of the cantilever and adjustment of the force acting between the tip and the sample [58].

## 3. AFM Imaging Modes

There are several modes for the imaging of the surface topography with AFM. The most commonly mentioned standard modes are as follows: contact mode, non-contact mode, tapping mode, and atomic force spectroscopy. The modes differ in the range of the interaction force between the AFM tip and the surface (short-range and long-range forces).

Contact mode is the simplest and the most widely used mode developed in AFM [59]. The cantilever tip is in constant physical contact with the sample surface. A feedback mechanism that allows interaction between the tip and the sample should remain constant during scanning. In contact mode, the tip of the probe is applied to the sample surface with a force of 10^−11^–10^−7^ N [60]. The cantilever tip constantly changes its position to maintain the same amount of deflection from the surface of the scanned sample. This mode is not particularly suitable for very soft surfaces, but much better results can be obtained for hard materials. The main disadvantage of operating in contact mode includes the susceptibility of the AFM tip to stick to the layer of surface contaminants that are present on the sample surface, which can result in image distortion [61].

In non-contact mode, the AFM tip oscillates at a frequency close to the resonance frequency (typically between 100 and 400 kHz) at a distance of tens to hundreds of angstroms relative to the sample surface. Here, the image is acquired with little or no contact with the sample. The sources of the attractive forces are the van der Waals interactions of atoms on the tested surface and on the surface of the tip [62]. This method is designed for the evaluation of very delicate materials. This mode has several advantages over other modes because the tip does not interact strongly with the sample. Moreover, this model is not subject to tip or sample degradation that can occur during “contact mode” measurements [63].

Positioning the tip close enough to the sample to make short-range forces detectable while also preventing the tip from sticking to the tested surface is the basis for another mode of AFM operations. Tapping mode is a key advancement in AFM. Very soft and delicate samples can be successfully imaged using this mode [64]. During scanning, the cantilever alternately touches the surface and rises at a frequency of about 50–500 kHz. This causes energy loss due to the periodic contact of the tip with the surface, so the amplitude of the vibration varies depending on the topography of the sample surface [65].

Atomic force spectroscopy (AFS) is very similar to contact mode. In this mode, the tip does not leave the surface at all during the oscillation cycle. This mode is used to image the flexibility of the sample and is ideal for imaging composite materials or soft samples on hard substrates, where contrast can be obtained between areas with different flexibilities [66]. AFS is a useful tool for studying adhesion forces, local elastic restoring forces, and local frictional forces [67,68].

## 4. Biomedical Applications of AFM

The first attempts to image cells using AFM began in the 1990s and involved dried red and white blood cells and Escherichia coli at outset, followed by dried Halobacterium halobium cells and living plant cells, and then red and white blood cells in buffer solution [69]. Further studies were conducted on mammalian cells, as they did not require additional processing (like dehydration, staining or coating) and fixation, but only immobilization, which did not affect the properties of the cells to any significant extent [29,69,70]. Subsequently, AFM started to be utilized not only to image cell surfaces but also to measure forces in biomolecular systems [26] and the nanomechanical properties of tissues [18,71,72]. In the late 1990s, it was possible to measure with AFM the forces required to break bonds between selected biomolecules, providing a basis for studying receptor–ligand systems [39,73]. These discoveries enabled further research on the intermolecular forces between antigens and antibodies [74,75].

The possibility of interaction between specific molecules has given origin to topography and recognition microscopy (TREC). This allows for the rapid identification of a specific receptor, the assessment of its location on heterogeneous biosurfaces, and the creation of a visual topography map [76]. In Chtcheglov’s study, the extracellular domains of VE cadherin were identified and localized on the surface of vascular endothelium using TREC [76]. This method may find application in oncology, as cell adhesion molecules (CAMs) have been shown to play a key role in tumorigenesis and metastasis [77,78]. CAM expression increases on malignant tumor cells, making their identification with AFM techniques potentially effective. However, studies to date have been limited by the low throughput and mapping time of cells in AFM [18,77,79,80].

Recently, there has been an increasing number of studies in the biomedical field comparing and correlating results obtained with scanning electron microscopy (SEM), transmission electron microscopy (TEM), AFM, confocal microscopy, Raman spectroscopy, and multiphoton microscopy (MPM). These techniques are complementary and are used for nano- to macro-level study. They provide information regarding morfological, chemical, and mechanical properties in order to facilitate a comprehensive understanding of the structure and properties of cells and biological molecules [81,82,83,84,85]. For example, differences between SEM and AFM images in the imaging of the liver’s parenchymal structures or ocular nerve fibers have been shown. Each technique revealed different details and subcellular structural information that correlated with each other. These were due to differences in the detection systems and sampling procedures of the two techniques [86,87]. By correlating different microscopic methods, more detailed information can be obtained and the scope of the study can be expanded [81,82,83,84,85,86,87].

The interest in the AFM technique in recent years can be seen in the number of studies available. The largest number of publications on the application of AFM in medicine available in PubMed are in fields such as ‘DNA research’ (approximately 3829 results), ‘Oncology’ (approximately 2910 results), ‘Blood cells and plasma protein’ (approximately 1541 results), ‘Immunology’ (approximately 1152 results), and ‘Neurodegenerative disease’ (approximately 830 results). A slightly smaller number of publications concerned ‘Aging’ (approximately 650 results), ‘Connective tissue’ (approximately 497 results), ‘Skin cells’ (approximately 339 results), ‘Hair’ (approximately 197 results), and ‘Melanoma’ (approximately 136 results).

### 4.1. Neurodegenerative Diseases

In the following years, the structure of proteins in the bound state and the dissociated state was studied [88,89,90,91]. For many years, AFM has been a very important tool for the study of neurodegenerative diseases associated with the deposition of peptide amyloid plaques and it has been the only instrument utilized for evaluating the structural dynamics of amyloid protein aggregates. Initially, Roeters et al. studied the process of the pathological aggregation of peptides and formation of amyloid fibrils [92]. Subsequently, the structure of β-amyloid and its oligomers [93,94], as well as the relationship between its formation and neurotoxicity [95], have been studied. Several authors have recently utilized AFM to assess the potential therapeutic mechanisms in neurodegenerative diseases, including Alzheimer’s and Parkinson’s disease [96,97,98,99]. Shao et al. used tetrahedral DNA nanostructures (TDNs) in their experiment to evaluate their effectiveness in inhibiting neural cell apoptosis. In this case, the AFM technique was used to confirm the correct morphology of the prepared TDNs particles. The authors showed that TDNs, by inhibiting neuronal apoptosis in Alzheimer’s disease, can positively improve memory and cognitive function [97].

### 4.2. DNA Research

AFM has also played a major role in DNA research. More modern AFM techniques, such as AFM biosensing, measure forces between complementary DNA strands or between oligonucleotides using highly specific building blocks [40,41].

There have also been important studies on the DNA mismatch repair (MMR) system using AFM. MMR plays a pivotal role during DNA replication and recombination. The mechanism protects cells from homologous recombination and mutations by signaling DNA damage. Therefore, by maintaining genome stability, it limits carcinogenesis in eukaryotic cells. Defects in the MMR system predispose individuals to certain types of cancer, the formation of birth defects, and infertility. Therefore, some authors studied the interactions between MutS, MutL, MutH, and DNA proteins in this system with AFM techniques [100,101,102].

### 4.3. Connective Tissue

Although AFM is mainly utilized in basic science, it has also found wider use in medicine in the diagnosis of cartilage tissue diseases. By examining the functionality of cartilage tissue, AFM may serve as a tool for the early diagnosis of osteoarthritis. Stolz et al. found the degradation of collagen, followed by the softening of the articular cartilage, and thus a reduction in the tissue’s nano stiffness in osteoarthritis. Initial changes can be detected only at the nanoscale and differ from those found in healthy tissues that undergo aging [72,103].

Osteoblasts and muscle cells represent other connective tissue cells that have been studied with AFM. Takai et al. analyzed the transduction of mechanical signals in osteoblasts by measuring cell stiffness and adhesion to various substrates [104]. Mathur et al. developed elastic moduli and studied the elastic behavior of cardiac and skeletal muscles [105].

The impact of osteoporosis on the appearance of bone tissue in AFM was assessed in animal models. AFM can evaluate the bone surface in 3D and the geometry of its microstructure, as well as the organization of the bone matrix and collagen fibers in regions with increased resorption [106]. In a recent study utilizing AFM measurements of adhesion forces, collagen fibers were found to swell in osteoporotic bones. This reduces the ability of collagen–apatite interfaces to catalyze the crystallization of hydroxyapatite. This was suggested by the authors of the study to be the crucial mechanism in the development of osteoporosis [107]. It is noteworthy that the aforementioned changes were identified using the AFM technique.

Bone marrow cells have also been of interest to researchers working with AFM. Recent studies have evaluated multipotent mesenchymal stem cells (MSCs), which have an important function in bone and cartilage regeneration processes. MSCs are of focus because they represent a potential source in regenerative medicine. To date, changes in stiffness have been studied and Young’s moduli have been developed for these cells. In addition, protocols for AFM measurements of MSCs during their osteogenic differentiation have been developed [108].

AFM has also been used to assess the biomechanics of cardiac tissues in arrhythmogenic cardiomyopathy, among others. It allows for the study of cell elasticity, adhesive forces, and viscoelastic properties. Therefore, AFM represents a promising tool with great potential for future research into fibrotic heart diseases [109].

### 4.4. Oncology

Recently, applications of AFM as a spectroscopic tool have expanded, especially in evaluating condensed soft matter or thin samples where other standard methods face significant limitations [110]. However, the refinement of this technique has found its most important applications in biomechanics for studying complex biological samples, such as cells and tissues, in terms of cancerous changes. Many biological processes taking place inside a living cell rely on the nanomechanical properties of cell substructures. AFM can provide information on the integrity and mechanical parameters of cell membranes. The high sensitivity and low force exerted on the sample make AFM a useful, non-destructive tool for studying cellular nanomechanics [111]. Vibrational spectra of neuronal cells and tissues obtained with AFM have made it possible to distinguish healthy and malignant brain tissues in real time [112].

It is worth noting that, with the use of AFM, it is not only possible to differentiate between healthy and cancerous cells, but the technique also makes it possible to distinguish the stages of cancerous conditions. However, the ability to distinguish cancer stages with AFM is still an area of active research and it is not yet fully established [113]. AFM cell imaging, supported by artificial intelligence (AI), can be used to precisely identify the phenotype of a cell. AFM is able to distinguish between two similar human colon cancer cell lines based on the levels of stiffness, which, as they increase, reduce tumor activity [114]. An analysis of specific surface features at the nanoscopic scale using AFM can distinguish pathways to the process of cell death (e.g., apoptosis, necroptosis, and ferroptosis). It allows the observation of distinct features of each regulated form of cell death. While performing mechanobiological elasticity analysis, it is possible to detect the early onset of cell death, which manifests itself as a significant decrease in elasticity [115].

The best-known group of anticancer drugs are cytotoxic substances. Because of their poor specificity, they can also cause damage to healthy cells and lead to a variety of adverse reactions [116]. Phospholipid vesicles and nanomaterials (e.g., lipid nanotubes) with biocompatible and biodegradable characteristics have attracted considerable interest for their potential use as controlled-release systems. Thanks to its ability to assess the physical properties and stability of liposomes, AFM is an effective technique that provides both morphological and metrological information on the properties of liposomes [117]. It is one of the most widely used SPM techniques, which provides information on the interaction between lipid carriers and active compounds, as well as data on the elastic, chemical and adhesive properties of the carriers. AFM also makes it possible to monitor the effect of drug treatments on the nanomechanical properties of solid tumors, which can lead to the identification of biomarkers [118].

### 4.5. Skin Cells

The study of skin cells made it possible to assess the cytoskeleton, the stiffness of cells in different layers of the epidermis, and the cytoskeleton’s differentiation and movement from the basal layer to the skin surface in order to evaluate the cell membrane lipids or nuclear mechanotransduction and to map the elastic moduli of the stratum corneum, epidermis, and dermis [119,120,121,122]. Modules of corneocyte elasticity and filaggrin, building a cornified cell envelope, were used to assess the mechanical properties of skin cells in atopic dermatitis [123]. AFM has also found application in studying the mechanical defects of cells caused by defective keratins and damage to the keratin network in blistering skin diseases, i.e., epidermolysis bullosa simplex [124]. By testing the forces of interaction between adhesion molecules, Vielmuth et al. conducted studies on desmogleins in pemphigus [125].

### 4.6. Aging

In dermatology, the effects of aging on skin and hair have been evaluated using AFM. Studies on skin fibroblasts, which are responsible for maintaining the mechanical properties of the dermal matrix, have shown that fibroblasts undergoing aging processes have reduced cytoskeletal tension and exhibit a considerable loss of traction force. This reinforces other factors associated with skin aging [126]. Other studies on skin photoaging have focused on collagen fiber content, which correlated with cell stiffness. AFM showed the reduced stiffness of skin cells subjected to long-term exposure to sunlight [127]. This parameter was evaluated in studies of drugs that exhibit anti-aging effects (i.e., retinol) [128]. Hairs also show differences in morphology during the process of aging. Jeong et al. showed that, after the age of 30, the diameter of the hair decreases, the surface of the scales is more undulating and rougher, and the edges of the scales are more broken. They evaluated all these morphological changes using AFM [129].

### 4.7. Hair

In terms of hair research, the physicomechanical and tribological properties of hair fibers, cells, and surface changes were studied. The research focus so far has been mainly concentrated on the effect of conditioners, hair-damaging external factors, and ethnic differences [130,131,132,133,134,135,136,137]. The latest AFM studies of hair utilized hybrid techniques. Among other things, spectroscopic techniques are being used in conjunction with AFM. Fellows et al. used AFM integrated with an infrared spectroscopy (AFM-IR) to study the chemical composition of the cuticle, medulla, and cortex of European hair [138,139,140].

In 2012, Shin et al. evaluated the morphology of the hair surface in patients with psoriasis vulgaris. They observed pits, increased roughness, and increased thickness of hair scales in patients with psoriasis. By imaging similar nanochanges within patients’ hair growing on unaffected scalps, they showed that psoriasis is generalized in nature [141].

### 4.8. Melanoma

Jeon et al. demonstrated that neoplasia modulates the mechanical properties of melanoma tissue. Representative stiffness maps and elastic modulus histograms were generated using force–distance curve measurements. It was found that benign pigmented nevi showed more stiffness than healthy skin. In contrast, melanoma tissues showed a wide variety of elastic modulus distributions, indicating a high heterogeneity of mechanical properties. In addition, the mechanical properties of all histological melanoma samples were independent of gender, age, anatomical location or Clark grade of invasion [142].

### 4.9. Immunology

The study of immune cell mechanics appears to be the new application of AFM. The functioning of the immune system is based on complex intercellular interactions, made possible by physical and mechanical forces between cells. To date, AFM has enabled the imaging and study of the formation of neutrophil extracellular traps (NETs), immune synapse formation between T lymphocytes and antigen-presenting cells, macrophage phagocytosis, and the process of membrane pore formation [143]. AFM enabled the real-time imaging of the changes occurring in neutrophils and its organelles during the NETosis reaction. By measuring the forces occurring in neutrophils, the steps indicative of chromatin decondensation and the release of NETs were characterized [144]. In contrast, when analyzing the interactions between T cells and antigen-presenting cells, Babak et al. studied the forces of interaction (adhesion) between the receptors that make up these synapses (complex TCR/CD3 and molecules LFA-1, CD43, F-actin) [145,146]. With the AFM technique, it was also possible to record height profiles and maps of cell membrane adhesion and pore stiffness in lymphocytes. The process of cell membrane remodeling under the influence of mediators such as perforins and gasdermins in tumor cells and the complement-induced membrane attack complex (MAC) in immune cells were visualized using AFM. In addition, the effects of the formation, remodeling, and repair of pores in cell membranes on the processes of cell death (apoptosis) were also studied [147]. Considering the above, one can conclude that recent discoveries are laying the basis for the new branch of medicine that mechanoimmunology is becoming [143,144,145,146]. A combination of the AFM technique and mechanoimmunology may contribute to the development of immunotherapy for blood cancers and perhaps solid tumors in the future [148].

### 4.10. Blood Cells and Plasma Protein

Previously, the morphology of red blood cells (RBCs), surface roughness, RBC surface antigen-specific biomolecules, and mechanical properties such as the architecture of the membrane skeleton and elasticity were studied [29]. Using Young’s moduli, changes in the stiffness and elasticity or deformability of the RBC cytoskeleton were shown [29,149]. Normal RBC cells were observed to have higher deformability. RBCs of patients with diabetes mellitus, hypertension, coronary disease, hereditary spherocytosis, thalassemia, G6PD deficiency, and sickle cell traits show a higher Young’s modulus value (i.e., greater stiffness) [150,151,152]. Differences in RBC surface roughness have been observed in conditions such as Waldenström macroglobulinemia, multiple myeloma, elliptocytosis, iron deficiency anemia, thalassemia, and diabetes mellitus, as well as during aging and in nicotinism [153,154,155,156,157]. The study of changes in RBC quantitative parameters was also used to evaluate the effect of oxygen concentration on the characteristics of RBCs in a model in vitro in states of normoxemia and hypoxemia. It was found that there is a specific state of imbalance between oxidative and antioxidant substances at a specific level of hypoxemia, which only minimally affects the disruption of RBCs [158]. However, testing RBCs’ values still requires the standardization of AFM parameters and sample preparation before AFM can serve as a diagnostic tool [29]. One of the most commonly studied hematological diseases with the use of AFM is malaria. With AFM imaging, it was possible to localize individual Plasmodium falciparum parasites in RBCs. AFM also made it possible to observe structural and adhesion changes in RBCs in real time at different stages of infection [29,159,160]. Further force spectroscopy research has opened the topic of targeted malaria drug development [161]. AFM is being used extensively to measure interactions between antibodies and plasma proteins (including fibrinogen) and RBC surface group antigens [29,162,163,164]. Some authors argue that the use of AFM in immunohematology could lay the groundwork for the development of highly selective and personalized medicine [29]. Also, the study of protein fibril aggregation on RBCs represents a new trend in the search for potential biomarkers of age-related neurodegeneration [165].

## 5. Limitations, Challenges, and New Trends for AFM Technology in Biomedicine

The main limitation of AFM as a diagnostic tool is that AFM cannot be used in the living human body. It is possible to examine cells in vitro from isolated cell cultures or ex vivo from biopsy samples [166,167]. Another limitation of the method is the very low experimental efficiency due to the low automation of the procedure and its time-consuming nature [116]. One of the limitations is also the technical preparation of the biological sample, so that the results obtained cannot be compared without prior standardization of the procedure in question. Depending on the preparation of the biological samples (cells may be fixed or unfixed but dried or resuspended in a specific solution), results may be different between studies and artifacts may affect the comparability of the images obtained [29]. It has been shown, for example, that the use of different cell preparation protocols affects different values of Young’s modulus [149]. In the future, sample processing and technical protocols for working with AFM (choice of probe tip and choice of appropriate equipment parameters) should be standardized for specific tissues or diseases between different research centres [29].

A new direction in biomedical research using AFM is the acquisition of spatial–temporal information. The introduction of high-speed AFM (HS-AFM) to study dynamic changes in biological molecules over time enables an in-depth analysis of the configuration of biomolecules without disturbing their function. It is a technique currently used to study the function of soluble and membrane-bound proteins, the organization of DNA chromatin, and viral replication [168,169,170,171]. In addition, another trend in improving the AFM technique is to combine it with other techniques. Hybrid AFM methods lay the groundwork for the targeted study of the chemical, physical, and morphological properties of specific structures that have been located at a nanoscale [1,17,48,139,140]. Currently, hybrid technologies combining AFM functions with other measurement techniques are the most promising. AFM-IR enables nanoscale infrared spectroscopy studies. Contact Resonance AFM uses the vibration of an AFM cantilever that resonates in contact with the sample for testing nanomechanical properties. Chemical force microscopy (CFM) extended the AFM technique to localize selected structures on the sample surface for chemical composition studies. AFM combined with confocal laser scanning microscopy (CLSM) correlates indentation points directly via fluorescence imaging of subcellular structures [10,116].

## 6. Conclusions

AFM evolved from the scanning tunneling microscope (STM), which was awarded the Nobel Prize in Physics in 1986 [19]. The STM used a conductive probe to electronically sense the surface of the conductive sample. Because of this, 2D images of biomolecules were unrepresentative and difficult to create [9]. AFM, based on the deflection of a scanning tip in contact or near contact with the surface under examination (without an electric field, as in STM), is more suitable for the visual testing of biological samples. The AFM technique is a future-oriented scientific tool thanks to its continuous improvement. Over the years, its ability to image cells or study their nanomechanical properties has evolved. Due to its highly complex nature, the study of biological samples was very complicated, requiring extensive knowledge in various fields and technical skills. In recent years, however, there have been significant advances and breakthroughs in the sophisticated study of biomechanical properties of human cells using microscopic techniques.
